# Thoracic Resection for Tumors Growing from the Bony Wall of the Chest

**Published:** 1900-03

**Authors:** 


**Thoracic Resection for Tumors Growing from the Bony Wall of the Chest.** By F. W. Parham, M.D. Pp. iv., 147. New Orleans. 1899.

Tumours of the chest-wall are not very common, but every now and again surgeons have to consider the advisability of their removal, and a careful study of the records given in this book will be of material assistance. The book is mainly a collection of records of cases, and hence the tendency to formulate rules of practice from insufficient facts is avoided; and if the surgeon will devote sufficient time to the study of the cases, he can form his own opinions as to the best way to deal with such conditions. But it would be a mistake to suppose that the entire value of the book lies in these records, for the author has ably summarised the main facts and drawn important conclusions from them.

Many of the records provide a most interesting study of the effect of freely opening the pleural cavity. In one case in which a growth of the sternum was removed, both pleural cavities were opened, but the patient recovered. Dr. Parham records two cases of his own. One of these is particularly interesting, as it shows that after collapse of the lung from free opening of the pleura, if the lung can be brought up and fixed, it will gradually expand and become functionally active again. Many of the cases have been taken from Mr. Stephen Paget's book on *The Surgery of the Chest*. There is one case of operation for cancer of the chest-wall, secondary to breast cancer. Few surgeons operate on such cases.

We must object to Dr. Parham's explanation of the production of pneumothorax. He says the pneumothorax pushes the mediastinum to the opposite side. If the pneumothorax consisted of a collection of air within the pleural cavity under pressure, as may exist in certain cases of perforation of the lung without external wound, then the mediastinum might be pushed over by the air: when one pleural cavity is freely opened, as in an operation on the chest-wall, the mediastinum is not pushed over, but when one lung is collapsed, it is drawn over by the elastic traction of the other lung.

Some very remarkable experiments on lung resection are referred to, and the subject of excision of portions of the lung in the human being is considered.

The work is certainly one of considerable value.

